# Poststroke Depression as a Factor Adversely Affecting the Level of Oxidative Damage to Plasma Proteins during a Brain Stroke

**DOI:** 10.1155/2015/408745

**Published:** 2015-03-08

**Authors:** Natalia Cichoń, Michał Bijak, Elżbieta Miller, Marta Niwald, Joanna Saluk

**Affiliations:** ^1^Department of General Biochemistry, Faculty of Biology and Environmental Protection, University of Lodz, Pomorska 141/143, 90-236 Lodz, Poland; ^2^Department of Physical Medicine, Medical University of Lodz, Plac Hallera 1, 90-647 Lodz, Poland; ^3^Neurorehabilitation Ward, III General Hospital in Lodz, Milionowa 14, 93-113 Lodz, Poland; ^4^Department of Toxicology, Faculty of Pharmacy with Division of Medical Analytics, Wroclaw Medical University, Borowska 211, 50-556 Wroclaw, Poland

## Abstract

Poststroke depression, the second most serious psychosomatic complication after brain stroke, leads to delay of the rehabilitation process and is associated with an increased disability and cognitive impairment along with increase in term mortality. Research into the biochemical changes in depression is still insufficiently described. The aim of our study was therefore to evaluate the possible association between plasma protein oxidative/nitrative damages and the development of poststroke depression. We evaluated oxidative/nitrative modifications of specific proteins by measurement of 3-nitrotyrosine and carbonyl groups levels using ELISA test. Additionally, we checked differences in proteins thiol groups by spectrophotometric assay based on reaction between DTNB and thiols. We also evaluated catalase activity in erythrocytes measured as ability to decompose H_2_O_2_. Correlation analysis was performed using Spearman's rank. We observed significant (*P* < 0.001) differences in all oxidative/nitrative stress parameters in brain stroke patients compared to healthy group. Our research shows that oxidative damage of proteins is correlated with the degree of poststroke depression, while nitrative changes do not show any relationship. We demonstrate a positive correlation between the concentration of carbonyl groups and the Geriatric Depression Scale and a negative correlation between the degree of depression and the concentration of -SH groups or catalase activity.

## 1. Introduction

In the past few years, notable attention is paid to mental disorders particularly prevalent among stroke patients, affecting about one-third of population. Physical disability and psychological stress occurring due to stroke need psychological treatment conducted together with physical treatment and rehabilitation [1]. Although affective disorders have not been formally established as independent risk factor for stroke and adverse prognostic factor responsible for impeding convalescing patients, attention to this physical/psychological symptom has continued to increase in the past decade [2]. A growing body of evidence acknowledges the existence of poststroke depression (PSD) [3].

Studies suggest that poststroke depression is the second most serious psychosomatic complication after brain stroke. Depression can occur at any stage after stroke (acute, subacute, and chronic), although some authors classify this condition as common mainly in the second phase of the disease [4]. Poststroke depression is associated with an increased disability and increased cognitive impairment and leads to delay of the rehabilitation process, due to the deterioration of motivation and low mood [5]. What is important in patients with PSD appears to be 3-4-fold increase in short- and long-term mortality compared with nondepressed patients [6].

However, research into the biochemical changes in depression is still not systematized and is lacking in both quality and quantity. Our aim in this study was therefore to evaluate the possible association between plasma protein oxidative and nitrative damages and the development of PSD in patients with acute ischemic stroke. In the past few years, oxidative stress has received much notice with regard to psychiatric illnesses including depression and it has been proposed as a contributing factor in the pathogenesis of depression [7]. Measurements of impaired redox balance after the ischemic stroke occurrence might be useful to monitor the outcome of patients who suffered an ischemic stroke in terms of stroke recurrence and other vascular events. This is particularly important for patients whose convalescence may be slowed down due to the presence of PSD.

Oxidative stress is thought to be a contributing factor in many chronic neurodegenerative pathologies, as well as acute cerebrovascular disorders such as stroke. The pathogenesis of acute ischemic stroke is highly complex and embroils multiple mechanisms. It is well proven that oxidative stress is involved in the pathogenesis of acute ischemic stroke. In ischemic stroke, blood flow is interrupted to a portion of the brain by a formation of clot blocking an artery. The later reperfusion of the engaged brain area when blood flow is restored is associated with a rapid increase in oxidative damage. Imbalance between the cellular production of free radicals and the ability of cells to defend against them is one of key mechanisms contributing to neuronal damage [8]. These conditions support oxidative and nitrative modifications in some amino acids. Proteins constitute one of the major targets of ROS/RNS (reactive oxygen/nitrogen species). Oxidative and nitrative changes in proteins include carbonyl groups formation, oxidation of the thiol groups and nitrotyrosine (3-NT) generation [9].

Some neurological disorders, including stroke, are associated with oxidative and nitrative modification of specific proteins and the accumulation of oxidative damage. There are several evidences that markedly indicate involvement of oxidative and nitrative stress in the pathophysiology of depression [10, 11]. Increased levels of ROS and RNS in depression, mainly peroxide [10] and NO [12, 13], and decreased levels of antioxidants, such as glutathione (GSH), in the postmortem depression brain have been demonstrated [14]. For this reason, oxidative and nitrative mechanisms have been proposed as targets for novel antidepressants [15].

Oxidative stress leads to inactivation and modification of antioxidant enzymes and weakening of antioxidant defense. The oxidation of proteins can be responsible for their conversion to forms more susceptible to degradation by proteinases [8, 16]. Protein carbonyl formation is an index of oxidative stress, as a result of amino acid modifications. Carbonylation of proteins is a nonenzymatic addition of aldehydes or ketones to specific amino acid residues, mainly to arginine, lysine, threonine, proline, cysteine, or histidine [17].

Shifts of redox-equilibrium in thiol-disulfide plasma system are implicated as a key mechanism of oxidative damage and restoration of redox homeostasis in postacute stroke patients. ROS-dependent oxidative modification of redox active cysteines within proteins is responsible for regulation of activity of a variety of protein functions, including enzyme activity, protein expression and abundance, protein localization, and interaction with other biomolecules in controlling of cell signaling and gene expression [18]. Oxidized cysteine residues can react with nitric oxide (NO) to form a nitrosothiol or with another protein thiol group to give a disulfide bond [19].

The nitrative stress can promote nitrosative posttranslational modifications of proteins that are implicated in the pathogenesis of the associated cardiovascular dysfunction [20, 21]. However, simultaneous formation of nitric oxide and superoxide radicals may reduce S-nitrosylation of protein due to concurrently generating the potent tyrosine-nitrating agent peroxynitrite (ONOO^−^). Protein nitration and accumulation of 3-nitrotyrosine is a biomarker of nitrative stress induced by peroxynitrite and other reactive nitrogen species. Tyrosine nitration as an usual modification is also a relatively specific marker of oxidative damage mediated by ONOO^−^, which is formed by the reaction of superoxide with nitric oxide and is important for the regulation of protein structure and function [22].

The antioxidant defense system in the organism comprises enzymatic and nonenzymatic components. In the pathogenesis of acute ischemic stroke low molecular antioxidants are consumed in the reaction with free radicals generated during the oxidative stress. The endogenous catalytic system includes a variety of antioxidant enzymes such as superoxide dismutase (SOD), catalase (CAT), and glutathione peroxidase. Among them, the first line of defense is a catalase which decomposes hydrogen peroxide, since its accumulation can lead to the formation of free radicals [23, 24]. The body of evidence suggests that depression belongs to the spectrum of (neuro)degenerative disorders and is accompanied by a decreased antioxidant status and by induction of oxidative and nitrative pathways. A significant association between depression and polymorphisms in genes involved in oxidative and nitrative pathways, like manganese superoxide dismutase, catalase, and myeloperoxidase, is also known [10].

In our study we compare the activities of CAT in the erythrocytes of PSD patients and nondepression patients.

Our study was aimed to investigate the dynamic of some oxidative and nitrative markers of plasma proteins during the convalescent of postacute stroke patients with depression.

## 2. Materials and Methods

### 2.1. Chemicals

Anti-dinitrophenyl (DNP) antibodies developed in rabbit, bicinchoninic acid protein assay kit, o-phenylenediamine dihydrochloride (OPD peroxidase substrate), and DNTB (5,5′-dithiobis(2-nitrobenzoic acid)) were purchased from Sigma-Aldrich (St. Louis, MO, USA). 2,4-Dinitrophenylhydrazine (DNPH) was obtained from Polish Chemical Reagents (POCH, Gliwice). All other reagents were of analytical grade and were provided by commercial suppliers.

### 2.2. Patients

35 patients following ischemic stroke were enrolled for the study and 29 poststroke patients were carefully selected who met inclusion criteria and did not meet any criterion for exclusion. A total of 35 patients (aged 74.3 ± 16.3) with moderate stroke severity (NIHSS scores 5.8 ± 3.5) who agreed to participate were included in this analysis. 29 patients were screened for positive results for depressive symptoms (GDS ≥ 11). Subjects with and without depressive symptoms were similar in demographics and clinical characteristics (Table 1). Patients with a medical history of prestroke dementia, hemorrhagic stroke, decreased consciousness, significant acute or chronic inflammatory factor or neurological illness other than stroke, and psychiatric disorder other than depression were excluded. All blood samples (control groups and patients) were taken in the morning (between 7 am to 9 am) in fasting status and stored using the same protocol. The protocol was approved by the Ethics Committee of the Faculty of Biology and Environmental Protection of University of Lodz, Poland number 16/2013. All participants provided written informed consent prior to participation.

Depression was screened using the Geriatric Depression Scale (GDS) on which a score ≥11 is a reliable and sensitive indicator of poststroke depression [13]. A trained psychologist researcher administered the GDS scale. The GDS, ADL, and NIHSS were administered either on the same day as the blood draw or on the afternoon before.

### 2.3. Determination of Protein Carbonyl Groups in Human Blood Plasma by ELISA Method

The detection of protein carbonyl groups was performed in human blood plasma using ELISA method described by Buss et al. [25] and modified by Alamdari et al. [26]. The microplates were incubated overnight at 4°C where plasma proteins were nonspecifically adsorbed onto ELISA plates. The wells were washed 3 times with 300 *μ*L PBS, reacted with dinitrophenylhydrazine (DNPH; 0.05 mM, 200 *μ*L, pH 6.2), incubated for 45 min at room temperature in the dark, and washed 5 times with 300 *μ*L PBS: ethanol (1 : 1, v/v) and a last time with 300 *μ*L PBS, all in accordance with Alamdari et al. [26]. Carbonyl groups were detected by anti-DNP antibodies (I step) and then a second antibody conjugated with horseradish peroxidase was added for quantification (II step) according to Alamdari et al. [26] procedure. This sensitive method was calibrated using oxidized albumin and required only 5 *μ*g protein per well. The standard curve was linear in the range of 0–3.36 nmol carbonyls/mg albumin [27].

### 2.4. Determination of Thiol Groups

Thiol groups in blood plasma proteins were determined using 5,5′-dithio-bis(2-nitro-benzoic acid) (Ellman's reagent, DTNB) [28]. The thiol-disulfide interchange reaction between DTNB and thiol is the basis of this spectrophotometric assay. The samples were mixed with DTNB and at the end of the incubation (1 h, 37°C) period the absorbances were recorded at 412 nm. The concentrations were calculated by using *ε* = 13600 M^−1 ^cm^−1^ and the results were expressed as *μ*mol/L.

### 2.5. Determination of 3-Nitrotyrosine in Plasma Proteins by the Competitive ELISA Test

The detection of 3-nitrotyrosine in blood plasma was performed according to the method described by Khan et al. [29] and was modified as was described previously [27]. The concentrations of nitrated plasma proteins were estimated from the standard curve, constructed with the use of 3-nitrotyrosine-containing fibrinogen (3-NT-Fg). 3-NT-Fg was prepared by the exposure of human fibrinogen to 1000 *μ*M peroxynitrite action. The presence of 3-nitrotyrosine in fibrinogen was confirmed spectrophotometrically at 302 nm, at pH 11.5 (molar absorption coefficient for nitrotyrosine, *ε* = 4400 M^−1^ × cm^−1^) [30], and then the obtained nitro-fibrinogen was used for the preparation of the standard curve, ranging from 10 to 1000 nmol/L of 3-nitrotyrosine-fibrinogen equivalent.

### 2.6. Catalase Activity Estimation

Catalase activity in erythrocytes was estimated spectrophotometrically using Beers and Sizer method [31]. Changes in absorbance were recorded at 240 nm. CAT activity was calculated in terms of nM H_2_O_2_ consumed/min using molar extinction coefficient *ε* = 36000 M^−1 ^cm^−1^ and shows as Bergmeyer unit [32].

### 2.7. Statistical Analysis

The statistical analysis was performed using StatsDirect statistical software V. 2.7.2. All values in this study were expressed as mean ± SD. The obtained results were analyzed under the account of normality with Shapiro-Wilk test. The significance of differences between the values was analyzed depending on the normality by unpaired* t*-Student or* U*-Mann-Whitney tests. Correlation analysis between obtained numerical data was performed using Spearman's rank correlation [33]. A level of *P* < 0.05 was accepted as statistically significant.

## 3. Results

**Figure 1:**
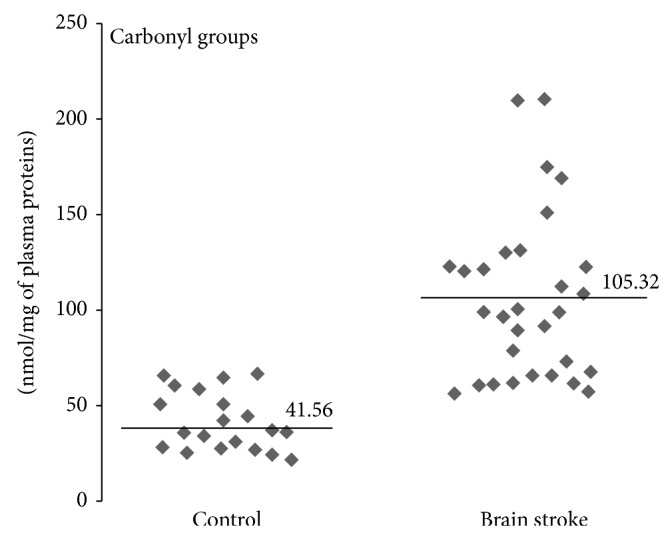
Differences of carbonyl group levels between stroke patients and control group. Carbonyl group level was measured in plasma proteins by ELISA method, *n* = 31, *P* < 0.0001.

**Figure 2:**
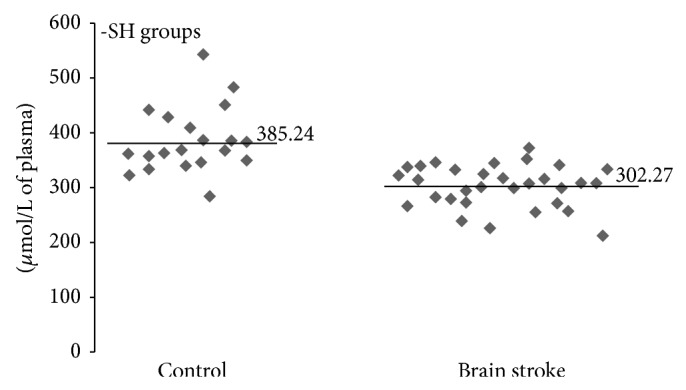
Differences of thiol group levels between stroke patients and control group. The concentration of thiol groups was measured in plasma proteins using Ellman's reagent, *n* = 31, *P* < 0.0001.

**Figure 3:**
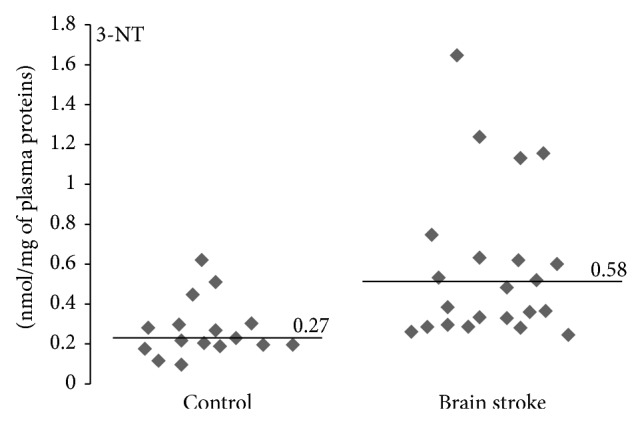
Differences of 3-nitrotyrosine levels between stroke patients and control group. 3-Nitrotyrosine level was measured in plasma proteins by c-ELISA method, *n* = 22, *P* < 0.0001.

**Figure 4:**
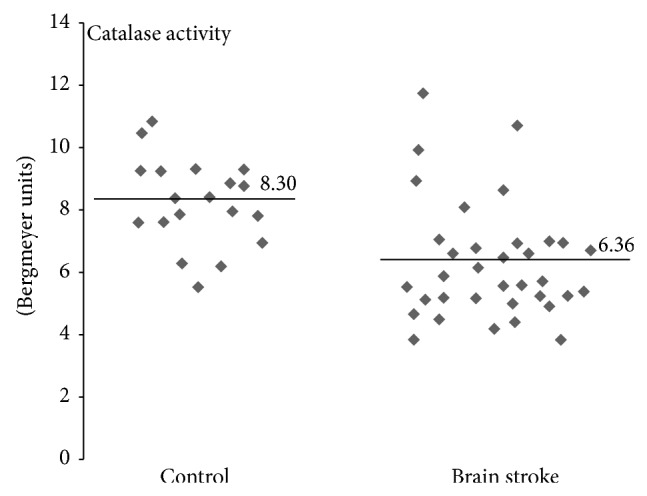
Differences of catalase activity between stroke patients and control group. Catalase activity was measured in erythrocytes using H_2_O_2_ disproportionation method, *n* = 35, *P* < 0.0001.

In our study we determined organism oxidative and nitrative stress parameters in stroke patients, especially in patients with poststroke depression. A comparative analysis shows that oxidative and nitrative stress parameters are significantly higher in stroke patients (both patients with PSD and nondepression patients) than in control groups (healthy people). All measured parameters were changed in stroke group (Figures 1–4). We observed a statistically significant increased level of carbonyl groups in plasma proteins (Figure 1), decreased level of plasma protein thiol groups (Figure 2), decreased catalase activity in erythrocytes (Figure 3), and increased plasma protein 3-nitrotyrosine level (Figure 4) in stroke patients in relation to control group. In the case of carbonyl groups and 3-nitrotyrosine, the observed increase in oxidative and nitrative protein damage after stroke was more than two times higher in patients than in controls (Figures 1 and 4).

**Figure 5:**
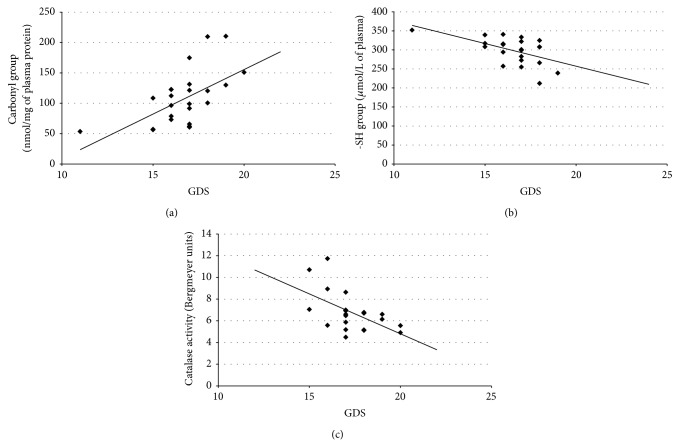
Regression plots of selected parameters of oxidative stress in patients with poststroke depression and GDS. (a) Plot for correlation between carbonyl group level and GDS; (b) plot for correlation between thiol group level and GDS; (c) plot for correlation between catalase activity and GDS.

Additionally, we observed dependence between changes in oxidative and nitrative stress parameters and poststroke depression intensity measured by Geriatric Depression Scale (GDS). As the distribution of the results of the GDS deviates from the normal, the method of Spearman correlation was more suitable for use in these analyzes. Obtained correlation parameters (Table 2) indicate the significant positive correlation between carbonyl group level and GDS, whereas between thiol group concentration/catalase activity and GDS we observed a significant negative correlation. For nitrative stress the marker which is a 3-nitrotyrosine showed no correlation with GDS. For dependence of carbonyl group level (Figure 5(a)), thiol groups level (Figure 5(b)), catalase activity (Figure 5(c)), and GDS regression plots were determined. We have shown the detailed course of these correlations, which confirms correlation and received numeric data presented in Table 2.

## 4. Discussion

The development of poststroke depression is a frequent mood disorder that affects around one-third of stroke patients; however, the etiology of PSD is not well understood. Probably, both the psychosocial and biological factors are significant in the development of poststroke depression [34].

Previous studies have identified relationships between PSD and increased functional disability [35, 36]. In the last studies poststroke depression was also associated with inflammation [37]. Previous studies have reported the elevated levels of some inflammatory mediators in PSD, including IL-18, [38, 39], C-reactive protein [40, 41], and IL-17 which is able to disrupt the blood brain barrier and contribute to neurodegeneration through increased production of reactive oxygen species [42, 43].

The brain with its high oxygen consumption and a lipid-rich environment is considered highly susceptible to oxidative stress or redox imbalances. Therefore, it is very probable that oxidative stress is implicated in several mental disorders, including depression. Although some studies have established a link between oxidative stress and psychiatric disorders, the causal relationship between them is not fully determined. Several mechanisms including genetic predisposition, abnormalities in traditional signal transduction pathways, and oxidative stress theory have been proposed to be involved in depression pathogenesis [7, 44, 45]. It has been demonstrated that depression in medically healthy patients has been associated with increased concentrations of blood cytokines [46], elevated levels of ROS and RNS [10–13], and increased F2 isoprostane excretion [47]. Moreover, considerably lower plasma concentrations of several key antioxidants, such as vitamin E, zinc, and coenzyme Q10, and reduced antioxidant enzyme activity, as well as reduced level of brain GSH, have been reported in major depression [10, 14]. The existence of a possible link between depression and central nervous system (CNS) inflammation is suggested in a few recent papers on the neuroimmunology of stroke [48, 49]. The mechanisms related with PSD may involve an imbalance between proinflammatory and anti-inflammatory activity responsible for augmenting of oxidative stress, which may weaken cognitive susceptibility. Recent studies demonstrate that oxidative and nitrative stress may contribute to the pathogenesis of depression through participation in neurogenesis/neuroplasticity, neuroinflammation, and monoamine reuptake process [50, 51]. Recent trend is that antidepressants exert a therapeutic effect by suppressing the production of inflammatory cytokines and ROS/RNS or increasing the antioxidant defense [15]. Therefore, inhibition of inflammation and concomitant oxidative stress may possibly contribute to the improvement of mental and physical efficiency of stroke patients. However, research into the changes and oxidative damage to major biomolecules in depression still lacks in both quality and quantity. Although the depression is one of the many consequences of stroke, it is still poorly diagnosed and inadequately treated [52]. Accordingly, these studies place particular emphasis on the examination of biochemical parameters that may directly affect the improvement of the functionality of patients depending on the severity of their depression.

Brain ischemia initiates a complex cascade of metabolic events, and most of them involve the generation of nitrogen and oxygen free radicals. Oxidative stress, associated with inflammation or postischemic reperfusion, may lead to oxidative and nitrative modifications of protein structure, leading to the alterations in their functional properties, and may cause accumulation of modified protein products, that has been observed under various conditions, such as aging, cell differentiation, and apoptosis [53]. The increase of oxidative stress parameters during a stroke is well documented in the literature. Oxidative stress plays a significant role in the damage associated with ischemic cascade arising from reperfusion, which is restoration of blood flow in the blood vessel after the hypoxia. Oxidative stress which occurs during a stroke significantly affects the irreversible neuronal damage [54–57]. After ischemia-reperfusion ROS may be generated from many sources, including an increased oxidation of catecholamines and hemoglobin, associated with a massive release of iron ions. Furthermore, ROS are produced due to changes in the mitochondrial electron transport, the activation of xanthine oxidoreductase and NAD(P)H oxidase, and during the metabolism of arachidonic acid [58]. As a result of hypoxia, a brain tissue is depleted of oxygen and glucose which are essential products for the ATP synthesis. Thus, it inhibits the activity of sodium-potassium pump and causes an inflow of calcium ions into the cell. Increased concentration of Ca^2+^ in the cell leads to the activation of Ca^2+^-dependent enzymes, including proteases and nucleases that are responsible for degradation processes of neurons [59].

Based on the above facts, the present study has been designed to explore the possible relationship between the level of typical markers of oxidative and nitrative protein modifications (carbonyl groups, 3-NT, SH-groups, CAT-activity) and the degree of poststroke depression. Oxidation of amino acid residues leads to the formation of relatively stable carbonyl groups that can be qualitative and quantitative markers enabling the assessment of the oxidative damage of proteins [60, 61]. In our studies, oxidative damage of proteins has been documented as an increased concentration of carbonyl groups in plasma proteins in patients after cerebral ischemia-reperfusion injury (Figure 1). Furthermore, a significant positive correlation between the level of carbonylation and Geriatric Depression Scale (GDS) has also been seen (Figure 5(a)). We have demonstrated more than twofold increase in protein carbonyl groups in poststroke patients as compared to healthy subjects. Our findings are consistent with previous reports. Wang et al. showed a significantly higher increasing the amount of carbonyl groups in the brain tissue of rats with hypoxia-induced brain damage [62]. The link between free radical generation and formation of carbonyl groups in protein molecules in different pathological conditions, including stroke [7], is well documented. However, our findings demonstrate for the first time a significant correlation between the degree of oxidation of the amino acid residues in plasma proteins and the GDS in post stroke patients.

The most important barrier that protects the brain tissue from damage is the blood-brain barrier (BBB). Particular role played the integrity of monolayer of brain capillary endothelial cells. Glutathione is low molecular weight antioxidant that protects endothelial cells from oxidative damage and affects the regeneration process after oxidative damage of cells occurring during a stroke [18]. Moreover, thiol compounds are the first line of defense against ROS and increasing of their oxidation is marker of oxidative stress [63]. Regulation of protein function through thiol-based redox switches plays an important role in maintaining redox homeostasis. Our study showed that concentration of thiol groups in plasma proteins in poststroke patients is approximately 20% lower than in the control group of healthy subjects (Figure 2). Analogous results are obtained by Tsai et al. and Wang et al. who studied the relationship between oxidative stress and the occurrence of other side effects in patients with acute stroke [64, 65]. Moreover, Tsai et al. postulated that the level of free thiol groups was significantly lower in patients with large-vessel disease than in those with small-vessel disease in the acute phase of stroke [64]. This proves that the level of free thiol groups depends on the extent of brain damage caused by oxidative stress, while our studies indicate that the degree of the thiol group oxidation in the plasma proteins is dependent on depression scale in poststroke patients. A significant negative correlation is indicated in Figure 5(b).

Protein nitration process resulting in the formation of 3-nitrotyrosine is mainly caused by strong biological oxidant peroxynitrite (PN) [66]. PN is a potent oxidant and nitrating compound, generated* in vivo *from the rapid reaction of two relatively less reactive but commonly found of free radicals: nitrogen monoxide (^•^NO) and superoxide (O_2_^•−^) [8]. Nitric oxide, a water- and lipid-soluble free radical, is generated by the action of nitric oxide synthases. Besides synthesis of nitric oxide, cerebral ischemia leads to the formation of the anion, mainly by the action of nitric oxide synthase, xanthine oxidase, and leakage of mitochondrial electron transport chain. There is increasing evidence that at high concentrations PN is the major compound responsible for ischemia-reperfusion injury or tissue damage by inflammation [67–71]. Ischemic stroke is an acute vascular event that obstructs blood supply to the brain and causes irreversible damage to both neurons and brain vessel cells. Immediately after the stroke, the ischemic tissue produces nitric oxide to restore blood perfusion but also produces superoxide anion. The interaction of these reactive species leads to the formation of peroxynitrite, which irreversibly nitrates protein tyrosines. Recent studies have emphasized the role of peroxynitrite in vascular damages. Increased 3-nitrotyrosine level was observed in various cardiovascular diseases [72]. Vadseth et al. reported the augmented level of nitrated tyrosine residues in plasma proteins derived from patients with coronary artery disease [73]. Bas et al. have demonstrated a significant increase in the concentration of 3-NT positively correlated with elevated activity of metalloproteinases activity (MMPs) in patients after ischemic stroke [74]. Similarly, increased concentration of NT-3 was observed in the studies Coucha et al. who investigated effect of neuronal damage during a stroke on the reactivity of cerebral vessels in rat. They confirmed the hypothesis that the loss of tone of blood vessel was affected by excessive production of peroxynitrite. On this basis, the authors postulated that ischemia-reperfusion injury impairs vessel reactivity via nitration [75]. Moreover, Tajes et al. who studied the effects of PN on the viability of the different brain cells also reported an important role of nitrooxidative stress in the pathogenesis of ischemic stroke [76]. Our studies have shown that the concentration of 3-nitrotyrosine in plasma proteins was approximately twice higher in poststroke patients in comparison with healthy controls (Figure 3). However, unlike in the case of oxidative stress, no relationship was found between protein nitrative damage and Geriatric Depression Scale.

Superoxide anion in addition to the formation of peroxynitrite is involved in a range of toxic mechanisms leading to tissue damage. The production of radicals in the brain is due to catecholamine metabolism such as dopamine and norepinephrine and is increased by the presence of transition metals and by a deficiency of antioxidant agents. Due to the high toxicity of free radicals the organism has developed a number of specialized and efficient methods to deactivate them. The superoxide dismutase enzyme (SOD) catalyzes a dismutation of the superoxide radical into hydrogen peroxide and oxygen. Afterwards hydrogen peroxide is reduced to water and molecular oxygen by peroxidase glutathione and catalase enzymes [77]. Catalase is not directly involved in the elimination of ROS but does not allow for Fenton's reaction to take place in which hydrogen peroxide is converted to highly toxic hydroxyl radicals. Following ischemia there is an increased enzymatic production of ROS, especially superoxide anion and hydrogen peroxide in a reaction catalyzed by xanthine oxidase. During reperfusion molecular oxygen is delivered and xanthine oxidase converts the hypoxanthine to xanthine. In such conditions superoxide anion is released in large quantities. It provides the opportunity for the production of hydrogen peroxide and consequently highly toxic hydroxyl radicals [59]. According to the literature, in stroke, the SOD value does not vary, while the CAT value decreases [77]. Reduced activity of catalase in stroke patients diminishes the effectiveness of deactivation of H_2_O_2_ [78], which can freely diffuse across the mitochondrial membrane, and therefore increases oxidative stress. In our study we noticed that that catalase activity in erythrocytes of stroke patients was about 25% lower in comparison with a control group of healthy (Figure 4). The similar effect of reduction in CAT activity in stroke-induced rats was also observed in studies conducted by Safwen et al. [54]. However, our studies have showed that decrease in the activity of catalase is dependent on Geriatric Depression Scale in poststroke patients. A significant negative correlation is indicated in Figure 5(c).

In conclusion, oxidative and nitrative stress parameters have been seen in the present study. However, only oxidative damage of proteins seems to be correlated with the degree of poststroke depression, while nitration of proteins did not show such a relationship. This indicates that the strategies attenuating oxidative stress in poststroke patients can be evaluated for efficacy as novel antidepressants in this population.

## Figures and Tables

**Table 1:** Clinical demographic characteristics. ADL: activity daily living; BMI: body mass index; GDS: Geriatric Depression Scale; NIHSS: National Institutes of Health Stroke Scale; NSAID: nonsteroidal anti-inflammatory; ASA: acetylsalicylic acid.

	GDS < 11 (non-D) *n* = 6	GDS ≥ 11 (D) *n* = 29	Healthy *n* = 20
Demographics			
Age (mean ± SD)	70.0 ± 14.6	74.3 ± 16.3	53.9 ± 10.6
Sex (% male)	66.7	47.2	25.6
Living alone (%)	48.2	56.7	33.8

Vascular risk			
Hypertension (%)	92.6	97.9	
Diabetes (%)	20.3	27.8	
Dyslipidemia (%)	65.8	76.8	
BMI ≥ 30 (%)	16.8	27.9	

Concomitant medications			
Antidepressants (%)	3.4	23.7	
ASA	62.8	78.8	
NSAID	8.7	20.8	

Stroke characteristics			
Weeks since stroke (mean ± SD)	5.3 ± 3.7	5.6 ± 4.2	
NIHSS scores mean ± SD	5.2 ± 4.7	5.8 ± 3.5	
ADL mean ± SD	11 ± 5.6	10.7 ± 6.7	

Lesion location			
Anterior (%)	12.0	15.8	
Posterior (%)	52.6	36.7	
Intermediate (%)	12.0	27.0	

Lesion side			
Left (%)	56.7	58.4	
Right (%)	43.3	41.6	

**Table 2:** Correlation coefficients values obtained for oxidative stress parameters and Geriatric Depression Scale. Correlation was analyzed using Spearman's rank correlation method and probability for correlation was presented in parentheses.

	Carbonyl groups	Thiol groups	Catalase activity	3-Nitrotyrosine

Spearman's rank correlation coefficient (Rho)	0.578941 (*P* = 0.0014)	−0.519103 (*P* = 0.0073)	−0.57903 (*P* = 0.0028)	0.071829 (*P* = 0.754)
